# Pretensive Shared Reality: From Childhood Pretense to Adult Imaginative Play

**DOI:** 10.3389/fpsyg.2022.774085

**Published:** 2022-02-28

**Authors:** Rohan Kapitany, Tomas Hampejs, Thalia R. Goldstein

**Affiliations:** ^1^School of Psychology, Keele University, Keele, United Kingdom; ^2^School of Anthropology and Museum Ethnography, University of Oxford, Oxford, United Kingdom; ^3^Department for the Study of Religions, Masaryk University, Brno, Czechia; ^4^Department of Psychology, College of Humanities and Social Sciences, George Mason University, Fairfax, VA, United States

**Keywords:** pretense, pretend play, games, imagination, table-top role playing, Dungeons & Dragons, pretensive shared reality

## Abstract

Imaginative pretend play is often thought of as the domain of young children, yet adults regularly engage in elaborated, fantastical, social-mediated pretend play. We describe imaginative play in adults via the term “pretensive shared reality;” Shared Pretensive Reality describes the ability of a group of individuals to employ a range of higher-order cognitive functions to explicitly and implicitly share representations of a bounded fictional reality in predictable and coherent ways, such that this constructed reality may be explored and invented/embellished with shared intentionality in an *ad hoc* manner. Pretensive Shared Reality facilitates multiple individual and social outcomes, including generating personal and group-level enjoyment or mirth, the creation or maintenance of social groups, or the safe exploration of individual self-concepts (such as alternative expression of a players sexual or gender identity). Importantly, Pretensive Shared Reality (both within the specific context of table-top role-playing games, and other instances) are primarily co-operative and co-creative. We draw on multiple examples, and focus on Table-Top Role Playing games (TTRPG) – and specifically, the most popular and enduring table-top role-playing games, Dungeons & Dragons (D&D) – as a primary example of such play. Our conception of “pretensive shared reality” links the widespread existence and forms of adult imaginative play to childhood pretense, places it within a developmental and evolutionary context, and argues that pretensive shared realities – which underpin many forms of imaginative culture – are an important topic of study unto themselves, and may be utilized to provide methodological insight into a variety of psychological domains.

## Introduction

It is received wisdom that adults do not engage in imaginative play, largely because the benchmark for this concept is childhood pretend play^1^. The likely cause of this assumption is the enduring legacy of Piaget (Göncü and Perone, 2005) who claimed that “*In a general way it can be said that the more the child adapts himself to the natural and social world the less he indulges in symbolic distortions and transpositions, because instead of assimilating the external world to the ego he progressively subordinates the ego to reality*” (Piaget, 2013, p. 54). Scholars following Piaget have also suggested that imagination tends to “go underground” (Harris, 2000; Singer and Singer, 2005; Piaget, 2013), that imagination transforms into other cognitive skills such as counterfactual thinking or daydreaming (Walker and Gopnik, 2013; Weisberg and Gopnik, 2013) or that it becomes subsumed by the consumption of fictional works in literature or film (Taylor and Mannering, 2007; Barnes, 2015). While Piaget is certainly a giant in the field of developmental psychology, his conception of development from pretense play to “games with rules” is, in our opinion, mistaken. We argue that “games with rules” ought not to be understood as “games with rules *and without imagination*.” We are not the first to make this argument, though we are in the minority for doing so (Lillard et al., 2010; Smith and Lillard, 2012; Weisberg, 2015), meanwhile, the vast majority of research on pretend and imaginative play has been conducted within the field of developmental psychology, with a particular focus on pre-pubescent children. Here, we extend this topic by examining pretense through an evolutionary lens to better conceptualize the purpose of imaginative play in adults. In so doing we better incorporate cross-cultural evidence and anthropological theory to better inform us of the purpose of imaginative play across the life-span, across cultures, and across socio-functional domains (Nielsen, 2012; Renfrew et al., 2017); we are also better able to draw connections between imaginative play and various domains of imaginative culture and belief, including religion (Renfrew et al., 2017) and strengthen a scholarly interplay between social and developmental psychology, anthropology, and a recently established niche within games studies which focuses on role-playing (Zagal and Deterding, 2018).

We will demonstrate that adults engage in sophisticated forms of imaginative play, and that this phenomenon is common and widespread (though our examples are principally WEIRD in nature). And, while imaginative play in adults frequently requires rules, it operates by the same cognitive mechanisms of imagination that support childhood pretense. Unlike childhood pretense, however, such play is – *critically*, possibly even *necessarily* – socially shared. We use the term “pretensive shared reality” to describe this broad capacity, outline what cognitive faculties we believe it relies upon, and discuss what the implications are of this phenomenon. Pretensive shared reality forms a frame of reference which is a “*product of motivated process of commonality of inner (mental) states with others about the world*” (Echterhoff et al., 2009). We will use the example of Table-Top Role Playing (TTRP) and Table-Top Role Playing Games (TTRPG) as a kind of case study, where we focus primarily on the two dimensions of operation of pretensive shared reality: physical embodiment and cognitive engagement. Our work is functionally an extension the “cognitive theory of pretense” advanced by Nichols and Stich (2000), inasmuch as we incorporate a necessary dimension of social sharedness and mutual representations that was initially under-developed. We conclude that pretensive shared reality plays an important role in imaginative cultures throughout the life-span and across many social domains that would otherwise be missed if we retained the parameters of “pretend play” as understood within the developmental literature. We ultimately argue that pretensive shared reality – as exemplified by table-top role-playing games – is an interesting topic of study in-and-of itself, and is potentially a valuable methodological tool for addressing challenging research questions in the field of experimental psychology. Finally, we will argue that our framework of pretensive shared reality re-casts the conceptual understanding of childhood pretense in a new light, allowing for new kinds of research questions to be generated.

Table-Top Role Playing Games appeared in their modern form in 1974 when the first edition of Dungeons and Dragons (D&D) was published (Ewalt, 2013). While D&D is among the most famous of the table-top role-playing game genre, it is by no means the only TTRPG, but its impact in popular western culture is ubiquitous. Even those who are unfamiliar with D&D as a game may be familiar with monsters such as *Beholders* (a floating spherical monster with multiple eyes on serpentine stalks), have consumed television or movies in which D&D is featured or central (the most recent example being *Stranger Things*), or have enjoyed the cultural output of writers and actors who credit D&D as being a source of inspiration, including George R. R. Martin, Robin Williams, Ta-Nehisi Coates, Felicia Day, and Stephen Colbert (Gilsdorf, 2014; Plante, n.d.). A summary description of how D&D is played is available in Appendix A for readers who are unfamiliar. And while table-top role-playing games generally, and D&D specifically, are relatively modern, the idea that individuals have been collaboratively and collectively creating and imagining alternative realities and the inhabitants thereof is likely as old as the art of story-telling itself. The basic cognitive concepts of role-playing – engaging in pretense that involves representing others’ minds in the first person (Lillard, 2001; Sachet and Mottweiler, 2013) – is familiar to anyone who has indulged the fantasies of a young child, or watched film, television, or theatrical productions. Table-top role-playing games (see Deterding and Zagal, 2018; White et al., 2018)^2^, then, are but the most recent and most adult-like instantiation of a tradition of fantastical, narratively driven, agentically rich expressions of imaginative culture that has roots in both the life-time development of humans, and the history of the species. We choose to focus on table-top role-playing as it is an highly illustrative example of adult imaginative play, and is a useful way to understand adult imaginative culture as the socio-cognitive extension of childhood pretend play (we have provided a glossary of potentially unfamiliar terms that are associated with table-top role-playing games, as we will use these throughout; see Table 1). While we trace and propose continuity between childhood play and adult-roleplaying, in the same breath it is necessary to acknowledge differences. The adult role-playing games are dependent on more complex social contract between participants and heavier ecologies of rules, which constrain in their aim of scaffolding the process of play (cf. Montola, 2008).

**TABLE 1:** Glossary of key terms and acronyms.

Term	Meaning
Player	The humans who are engaging in creation and maintenance of the pretensive shared reality.
Player-character	The players’ representative in the pretensive world, constructed according to a set of rules and operated by the player. In principle, while the player knows the mind of the player-character, but player-character must act as if it has without awareness of the mind of the player.
Game master (GM)	A unique player within the genre of table-top role-playing games who primarily regulates what is legal within the pretensive shared reality. The GM serves two roles – storyteller and referee. The first is in determining and describing relevant features of the pretensive world that the player-characters inhabit. The second is in determining what actions are consistent with the rules of the table-top role-playing game system. The GM does not operate a player-character, and is not responsible for regulating the actions of [players’] player-characters. A GM may ‘set the scene’, but it is the players who – via their player-characters – “direct the action.”
Non-player character (NPC)	A pseudo-player-character operated by the GM. NPC’s are independent from players and characters, but exist to populate the world and enrich it. For example, if player-characters are working for some kind of leader – who is not one of the players’ player-characters – then the GM must create the leader, providing description, dialog and a set of ascribed motives, beliefs, and actions.
Table-top role-playing/table-top role-playing games	Table-top role playing/Table-top role playing games. At its core, a table-top role-playing game is a set of rules and mechanisms that allow for coherency within the pretensive shared reality for a small group of players “around a table.” The most famous example is Dungeons and Dragons (D&D), though countless variations and alternatives exist.
Pretensive shared reality	Pretensive shared reality describes how a group of individuals employ a range of higher-order cognitive functions to explicitly and implicitly share representations of a bounded fictional reality in predictable and coherent ways, such that this reality may be explored and invented/embellished in an *ad hoc* manner to the semantic and narrative benefit of the group, and in so doing, facilitate social utility.
Social utility	Social utility is the purpose for engaging in pretensive shared reality. While the specifics may vary from player to player it may include generating personal and group-level enjoyment or mirth, the creation or maintenance of social groups, or the safe exploration of individual self-concepts (such as alternative expression of a players sexual identity). This term is deliberately broad and should not be regarded as a prescriptive definition, but simply a place-holder term to describe the variety of motives present for engaging with TTRPGs in a social context. Our definition can be abstracted and reduced to the following: Social Utility, under the umbrella of pretensive shared reality and table-top role-playing is normatively co-operative, co-creative, and not interpersonally competitive, and contains a heterogenous set of behaviors that can be dis-aggregated in meaningful ways.
Imaginative cultures	The present manuscript is part of a special issue focusing on “imaginative cultures.” We accept the provided definition of the term: Imaginative culture consists in shared and transmissible mental experiences that are aesthetically and emotionally modulated.
Physical embodiment, axis of	We define “embodiment” as the degree to which the pretensive shared reality (or imaginative culture) *requires* the participant to perform actions which are externally visible and behaviorally representative of, and consistent with, the pretensive shared reality with their physical body
Cognitive engagement, axis of	We define “cognition” as the degree to which the experience *requires* cognitive engagement that is consistent with the object of shared intentionality (i.e., the pretensive reality of the group)

## Imaginative Play as “Pretensive Shared Reality”

We use the term “pretensive shared reality” to capture the psychologically rich and complex phenomenon of a specific kind of imaginative culture. In doing so we must take care to clearly define our terms. We use “pretensive” to describe something that is derived or maintained by the faculties of imagination, which differs from the reality of one’s perceivable environment (that is, a reality that is different from the “real” world). The term “shared” has two meanings. The first is dynamic: it is a process in which multiple individuals engage with each other with shared intentionality (Rakoczy, 2007), and the second is that the “shared reality” is, to some degree, institutional or normative, and that the object of shared intentionality has a set of premises, assumptions, and semantic features that are durable for the period of group activity, and which may endure beyond the immediate period of shared intentionality (e.g., over multiple instances of game play). We will elaborate on the term “reality” later with regard to concepts described by Nichols and Stich (2000), but at its simplest, “reality” should be understood as the details of the imagined “workspace” that individuals collectively share, react to, influence, and elaborate upon. Thus, pretensive shared reality describes a constellation of mental objects subject to shared intentionality, which is durable, and which embodies a set of (implicit or explicit) premises, rules, or norms, that knowingly differ from the “real” world by some degree. Finally, pretensive shared reality permits and constrains the behavior of both players and their player-characters (consistent with the premises and/or norms of the imagined world). While our term captures the process and outcome of actors on a stage^3^, “pretensive shared reality” is more useful as it also captures play between children, as well as the experience of table-top role-playing, and includes more impoverished imaginative cultures such as fancy dress parties, fantasy sports leagues, and cultural rituals like Christmas or Halloween. Our term also excludes imaginative cultures associated with the production or consumption of fiction: while we do not deny that writing a fictional story is imaginative, and may even be collaborative, we do not regard it as a form of “play;” simultaneously, reading a piece of fiction (or observing one performed on stage), while moving and often social, does not require the consumer to perform actions consistent with the premises of the imaginative object (meanwhile, the actors *are* engaging in a pretensive shared reality as they are *required* to act in certain ways).

Importantly, our conception of pretensive shared reality emphasizes the continuum of adjacent concepts of imaginative cultures, which include various manifestations of the performing arts, hobbies associated with counter-factual realities, and conceptions of wild and institutional religions (Whitehouse, 2004; Renfrew et al., 2017; Boyer, 2020). In psychological terms, pretensive shared reality generally, and table-top role-playing specifically, draw upon many higher-order cognitive faculties. These include Theory of Mind, the ability to entertain that the contents of another’s mind is different from one’s own (Ma and Lillard, 2017; Wellman, 2018); metarepresentational abilities, that a stick may be regarded as a sword (Kim et al., 2021), pretense (Lillard, 2017), the ability to imagine things that are not real (Shtulman, 2009); norm psychology (Rossano, 2012), shared intentionality (Rakoczy, 2007), and many others. Beyond individual cognitions, the specifics of any individual table-top role-playing situation requires that the players *share* the same understanding, and can manipulate their *player-characters* within the situation that is consistent and coherent. While we recognize that – as people – were we to jump from a 10th story window we would likely die, we can negotiate and hold a shared understanding of a pretensive reality where, if our player-characters were to do the same, they might float gently to ground. The specifics of the example are less important than the mere fact that we, as adult humans, can entertain the premise of, and sensibly determine the consequences of, certain imaginative statements, where such statements may be counter-intuitive or even impossible. Thus, the term “pretensive shared reality” encapsulates a host of higher-order cognitive functions that can be explicitly and implicitly shared by a group of individuals to represent a fictional reality in predictable, coherent, and unlikely ways, such that the “reality” can be explored, expanded, and driven in directions that endure and enrich.

Our definition of pretensive shared reality has similarities with the work of Nichols and Stich (2000), who describe the cognitive architecture of the “pretend world box” and the phenomenon of “cognitive quarantine” as well as describing the four elements that constitute pretend play (in adults): establishing a premise, inferential elaboration, non-inferential elaboration (embellishment), and production of appropriate pretend behavior. Their work is rightly well regarded, but as we will argue, it doesn’t go far enough. For example, they claim that *“there are surprisingly few examples of adult pretense described in the psychological literature*” (p. 118), and then describe how they asked student volunteers to pretend in various scenarios, including pretending that a banana is a telephone, and asking dyads to pretend to be a waiter and a diner in a restaurant. What they fail to include in such illustrations is that, even in the first instance [of the banana] there is a *shared* quality to the pretense. It is as if the experimenter is saying *“I propose the premise that the banana is a phone, and I would like you to accept this premise and act in a manner consistent with it*.” In practical terms, this is no different from a Game Master (GM; see Table 1 for a definition) describing a scenario (*You stand in an otherwise empty room. In front of you is a table, and on that table is a phone)* and asking a player what they want to do. No instructions are given for how to operate in either context: in both cases the student volunteer or the player could call the police for help, order a pizza, or pretend that someone is calling them. Similarly, the scenario of the diner and the waiter in the restaurant is functionally no different from two players (and a GM) pretending to occupy the roles of a fighter and a healer in combat, i.e., the roles clearly signify a space of social and functional affordances for all parties. We argue that adult pretend play is not surprisingly infrequent, it just comes with a specific set of institutions; in the case of table-top role-playing games these institutions are a rule book, and a GM. That said, the concepts of the pretend world box and of cognitive quarantine posited by Nichols and Stich are extraordinarily illuminating for the task at hand.

Now we must consider “cognitive quarantine” – and for the sake of consistency merge it with the TTRPG terminology (see Table 1) and ethnography. It is obvious that the actions or beliefs of the *player-character* have a limited effect on the *players* and *their [real] world.* The player is not changed if their player-character eats, gets rich, falls in love, or pursues any other in-world experience. The play is cognitively quarantined and creates a safe creative zone, which Nichols and Stich would probably perceive as fully separated from the player’s world. However we must add that at least one of the strong motivations of playing role-playing games is the aesthetic^4^ and emotional affection from the play to the player (Stenros and Bowman, 2018). Consider the case of player-character death. Nothing in the real world has died, but the experience of grief may be authentic: the player can be moved by the death of their player-character due to the affective or aesthetic connection – how then, does cognitive quarantine account for such experiences?

We suggest that the same set of phenomena which Nichols and Stich call cognitive quarantine has its own history under the umbrella term of “magic circle” or “boundary of play” within games studies (Stenros, 2014), where it is in some ways more elaborated due to the more variable range of experiences. We presume most challenging topics for any theory of pretense will be connected with the question of permeability of the boundaries between the play and non-play, which is especially relevant for embodied role-playing. We agree with Nichols and Stich’ thesis, that within the pretend-world-box that cognition mostly operates in the same way as outside (no concept of special pretend cognition is needed), nevertheless they somewhat neglect a feedback effect of pretending experiences, the [pretenders’] ability to form living streams of experiential states, in which players are not just producers maintaining two disparate levels of communications, but also recipients, as well as objects of pretense upon which others can act. The cognitive theory of pretense does not seem to take into account the problem of affective states (see the concept of *immersion* in the role-playing studies, Bowman, 2018) of the real person and its relation to the pretend-world-box of the player-character, or the necessity to discuss the cognitive-affective distribution of cognitive and affective resources for pretending. The porousness of the boundary of the play can be illustrated best through spillover effects of play into player, or vice versa, in so called “bleed-effects” (Montola, 2011; Leonard and Thurman, 2019) – an experience where cognitive qualities of the player may *bleed-in* to the player-character (such as a player’s fear of spiders unintentionally influencing an player-character’s action), or where the cognitive qualities of the player-character may *bleed-out* into the player (such as a player’s sincere feelings of grief). Thus, even though these experiences are partially quarantined (i.e., the player still recognizes the source of the experience), they also blend experiences, which posits challenging examples of inadequacy of any pretense quarantine theory with strict boundaries.

We find the representational theories of pretense and the work of Nichols and Stich (2000) still pragmatically relevant for elaboration of cognitive theory of pretense, but in respect to adult role-playing we find it is useful mainly if we omit the affective dimension and think of the problem as a question of an socially mediated, unconstrained creative space dealing with information exchange and maintenance. Here, Nichols and Stich’s concepts of cognitive “boxes” that facilitate pretense remain useful. First, there is the “Possible World Box,” which is simply the “workspace” of imagination, a set of premises and semantic claims about the world for the players in which the player-characters are operated. In table-top role-playing game terms, the possible world box is simply “the game world,” and the game world is largely described and operated by the GM, though player-characters can directly influence the game world (e.g., if their player-character kills the Evil Queen who is holding the Beautiful Dragon captive, then the game world no longer contains the Evil Queen). Nichols and Stich (2000) go further, arguing that there is an updating system, and that the player has a “belief box,” or a set of belief-like representations consistent with the possible game world. The important thing about pretensive shared reality, and the very concept of adult imaginative play^5^ and imaginative culture, is that pretensive shared reality does not require perfect fidelity in sharedness, simply shared intentionality (Rakoczy, 2007). By sharing and distributing cognitive obligations to maintain the [shared] pretensive reality, the imaginative world becomes more dynamic. The GM is responsible for regulating the “official” game world (the possible world box) and holds in mind simple belief boxes for NPCs (i.e., the motive of the Evil Queen), meanwhile, the players hold a (incomplete but functional) “belief box” about the game world, and hold elaborate and rich belief boxes of their own player-characters. Players (including the GM) and their player-characters (including NPCs) can hold belief-like concepts within their belief box about other player-characters and the game world generally. But so long as the premises are maintained, as described and regulated by the GM, engagement with the pretensive shared reality can generate a nearly limitless set of imaginative and creative expressions from an initially constrained set of circumstances.

## Why Do Adult Pretenders Pretend at All?

Having established that a central feature of pretensive shared reality is that it is *shared* by a group, we can address a question that Nichols and Stich (2000) struggled to answer: *why do [adult] pretenders pretend at all?* While they labor on multiple simplistic examples, primarily revolving around why adults would engage in pretend play with children – for example, when representing a train, one may cycle their arms and say “Chugga chugga choo choo” – we assert that pretensive shared reality, whether between a child and an adult, or a group of adults, is to facilitate *social utility^6^*. We use this term to refer to the manner in which pretensive shared reality *facilitates* particular outcomes, and the *functions* that can be attributed to it; social utility encapsulates cooperative behavior (that is rarely if ever intentionally interpersonally competitive), which may generate personal and group-level enjoyment or mirth, the creation or maintenance of social groups, and/or the safe exploration of individual self-concepts (such as alternative expression of a players sexual or gender identity). This is true whether a father is pretending to be a tiger while chasing his young child, or whether four 35-year-olds operate invented player-characters to free the Beautiful Dragon from the Evil Queen.

An important additional element of social utility, and one of the reasons we speculate that adult pretenders pretend as they do, is that pretensive shared reality in the form of table-top role-playing games are expressions of alternative, unreal, or impossible agencies. At first glance, the inclusion of “agency” as a motivating feature of social utility within the context of pretensive shared reality may seem out of place. However, we believe it’s inclusion is a necessary extension of the work of Nichols and Stich (2000), and incorporates other anthropological research. First, Nichols and Stich (2000) do not explicitly address the issue of agency, though it is implicit throughout their 2000 publication (and apparent in some subsequent publications, particularly those of Nichols, 2000, 2004; Nichols and Stich, 2003), and is particularly relevant to their fourth condition of pretense (regarding behavior). Second, recent philosophical scholarship places agency as an under-appreciated concept within the study of “games.” Furthermore, agency as a necessary component of pretensive shared reality (and consequently an important motivation within this framework) is what separates pretensive shared reality from more passive imaginative cultures, and more linear forms of play (such as computer games). And third, while most people engaging in pretensive shared reality are doing so with minimal personal investment, some individuals and groups engage in profound explorations of agency and concepts associated with expression of identity and morality (among many others). Consider the case where individuals are safely exploring alternative gender or sexual identities that are not presented to the “real” world (a point which we expand upon with our discussion of Turner’s (1969) concept of *communitas* and *anti-structure*).

Nichols and Stich (2000) only implicitly addressed questions of agency. While children may engage in pretense spontaneously (thus, within children there is agency in both the content of the pretense and the premises thereof), adults do not (at least, not in the same way as children). Nichols and Stich (2000) report a set of pretense scenarios for one individual and for dyads (note: in both cases the experimenter acts as an additional agent in these scenarios, that of the premise-setter). The solo examples involve pretending a banana is a phone, that the individual is a train, that they are a dead cat, and that they are “home alone at night and [you] hear a suspicious noise in the basement.” Each of these is a very lab-friendly scenario, which are superficially open-ended, but which actually only permit a relatively constrained range of agencies. Each scenario has situational affordances^7^ which are constrained by unacknowledged and unstated premises relating to the intention of the researcher. The “noise” scenario is the most open ended, and individuals may conclude it was a burglar and consequently acted consistent with this (fighting, fleeing, or calling the police); or they may have regarded the noise as something harmless, created by the wind or a stray cat (hopefully one that is not dead). Of course, other verisimilar options exist, but for the sake of the argument they are not relevant. In any case, the player was implicitly directed by the situational affordances to *resolve* the mystery aspect of the premise, embellish it, and act. However, it seems unlikely that any participant went against this implicit demand. The premise was not “*You live in a world where anything is possible, and right now you are hearing a noise in the basement*….” If so, someone might have justly responded “*I sprinkle fairy-dust on myself and fly to Never-Never land and spend the evening drinking Pan Galactic Gargle Blasters with Sherlock Holmes and Cleopatra*.” No, the implicit instruction (and stated affordances) were to resolve the noise. Agency, then, was highly constrained. This is also true with the dyadic instructions, which were both based around a server-and-diner in a fancy restaurant or a fast food restaurant. The implicit demands were to imagine specific kinds of food, and mannerisms. It seems unlikely any dyad play-acted something like the scene from *Pulp Fiction* where a diner decides to rob a restaurant, even though such an action is well within the realm of possibility. Again, agency is constrained by task demands and existing schemas. Pretensive shared reality is constrained by situational affordances by design, but in the case of TTRPGs, their defining feature is their open endedness (in such games, flying to a new location, or robbing the restaurant, while possibly surprising, would be wholly acceptable). Nichols and Stich (2000) imply that agency is important by indicating that individuals may creatively embellish a situation, and act consistent with the premises and all subsequent information, but do not go so far as to state that such decisions *require* agency. And yet, it is clearly the case that agency is a requirement, and that any examination of adult pretense or imaginative play without this consideration is examining only an impoverished subset of the phenomenon.

Beyond addressing the absence of agency within a useful theoretical framework, there are other reasons to consider it more closely. Nguyen (2020) argues that, just as paintings are a medium of vision, music is a medium of sound, and stories are a medium of narratives, that games are a medium of agency. The ways in which we interact with games – and in this case, table-top role-playing games – allows us to experience, master, and derive pleasure from forms of agency not available to us in the real world. While other modalities of experience – such as reading or watching a movie – can transport us, can influence our cognitions, our affect, and our sense of the world (Green et al., 2004; Brown, 2015), so too can games. The key distinction is that pretensive shared reality requires behavior on the part of the players (even if that behavior is as simple as decision making and speech acts). Engaging with a pretensive shared reality allows individuals to collaboratively co-construct a place for agencies to take place, where these agencies can take on forms from the trivial to the profound. As stated, there is no *a priori* reason to limit oneself to ordering food in a fantastical restaurant (even impossible foods); one may do anything, and that includes armed robbery. Pretensive shared realities, specifically in the form of table-top role-playing games, may produce novel cognitive content, and affective or agentic experiences which may be valuable to the players as individuals or as a group (or both). The opportunity for deeper involvement in the medium – via agency – is principally greater than in more passive artforms that do not require behavioral engagement.

Finally, having established established pretensive shared reality – specifically in the form of table-top role-playing (but certainly not limited to it) – is generally an attempt to provide many degrees of agentic freedom, and that that agency is relatively unique to such games (but again, not entirely limited to it), we may consider the anthropological work of van Gennep (1909); Turner et al. (2017) and a discussion of *communitas* and *anti-structure* in the context of transformative rituals. While we are not suggesting that TTRPGs are akin to rites-of-passage, some similarities exist. Communitas and anti-structure may be regarded as a phenomenon in which members of a community temporarily suspend ordinary regard for social structures, conventions, and existing status hierarchies (*anti-structure*) and enter into a new social context in which all individuals are equal, with the express purpose of being able to participate in, and share, a specific common experience (*communitas*) by “*separating*” (p. 94) from their “*states*” (ordinary social roles and schemas). In the context of TTRPG this may be seen when adults play the role of children, assume alternative genders, eschew their real-world credentials (e.g., lawyers, professors, doctors), and ignore (or even reverse) real-world status-hierarchies (e.g., employee and employer). The players then enter a “*liminal*” space: the game-world (or rather, given that no full transition between states occurs, it should be regarded as a “*liminoid*” space; Turner, 1974). In TTRPG terms, the “*characteristics*” of the players and associated player-characters are “*ambiguous*,” they exist in “*a cultural realm that has few or none of the attributes of the past*… *stat*e” (p. 94), and liminal entities [players and player-characters] “*are betwixt and between the positions assigned and arrayed by law, custom, convention, and ceremony*” (p. 95). The communitas generated in this process is one of:

…*homogeneity and comradeship*… *a “moment in and out of time” and in and out of secular social structure which reveals, however fleetingly, some recognition (in symbol if not always in language) of a generalized social bond that has ceased to be and has simultaneously yet to be fragmented into a multiplicity of structural ties* (p. 95).

Put in more contemporary terms: players sit at a table, and engage in an extended period of social egalitarianism, where they reduce themselves to alternative social-forms (their player-characters), and participate as equals in a common, shared experience. However, the analogy ought not be stretched too far. Turner (1974) and others argue that after this liminal phase, individuals are “aggregated” into new roles (i.e., a boy becomes a man, a girl becomes a woman, a neophyte becomes an acolyte) and they return to the “real” world in a new state. This is obviously not true in the case of role-playing games – such hobbies fall short in many dimensions of the richness of rituals – but this doesn’t mean that the experiential quality of eschewing social roles to engage in pretensive agency in an equitable manner with like-minded others cannot be regarded as a meaningful, and even symbolically potent, experience^8^. Importantly, unlike rituals, one may construct their *liminal personae* (p. 95) (rather than have it assigned by doctrine or belief) in order to express agency and co-construct the pretensive shared reality. This experience of seperation and liminality, of anti-structure and communitas – despite falling short of full aggregation/transformation – facilitates what we are referring to as social utility: [the] generation of personal and group-level enjoyment or mirth, the creation or maintenance of social groups, or the safe exploration of individual self-concepts (such as alternative expression of a players sexual identity).

Thus, our response to the question *why do adult pretenders pretend at all?* is simply that adults are motivated to engage in imaginative play as it facilitates social utility, which includes the expression of alternative agency – agency which is *necessary* for a pretensive shared reality to be co-constructed and maintained, agency which is *unavailable* in other domains of real life, and agency which may be *explored or exercised* without regard to real-world social norms and structures.

However, the question of “*why adult pretense*” has at least two levels of analysis. The above-provided answer relates to the *proximal* interpretation, while the *ultimate* interpretation remains unaddressed. The ultimate level of analysis forces us to ask why adult pretense exists *at all* (Tinbergen, 2010; Bateson and Laland, 2013). Let us first consider what childhood pretend play is, then to examine why childhood pretend play is thought to exist, before coming to a conclusion on why adult pretense exists at all (and is not simply left behind, as Piaget suggests).

## Childhood Pretend Play

Childhood pretend play is seen in typically developing children across cultures and contexts (Haight et al., 1999; Lillard et al., 2010). While definitions vary, pretend play is most often characterized by non-literal actions produced for non-instrumental purposes such as enjoyment and exploration (Weisberg, 2015). No single behavior is indicative of pretend play, rather, any one of a suite of behaviors are typically characterized as pretense. These include object substitution (e.g., using a pencil as a rocket ship), attribution of properties (e.g., pretending a doll’s face is dirty, and cleaning it), role play (e.g., running around as a superhero), and pretense metacommunication (e.g., discussing the rules and setting up an imaginary dentist’s office; Thompson and Goldstein, 2019). Thus, pretend play can involve both social and non-social representational content (Sachet and Mottweiler, 2013). Pretend play is active and embodied most of the time (and is mostly measured through physical action), although it does not have to be (and can occur only in a child’s mind), however, active physical embodied is most common.

The earliest instances of pretend play is quite simplistic (occurring in children as young as 12–18 months). Typically it is a free-form solo activity without shared intentionality, and does not contain normative rules or fantastical elements. As children move through the preschool years into early elementary school, pretend play moves from *ad hoc* counterfactual social scenarios (that typically do not endure across multiple times or contexts) to durable preplanned counterfactual worlds, explicitly incorporating normative rules, social intentionality, and collective negotiation (Fein, 1981). Engagement in pretend play also varies widely in how grounded it is to reality and everyday actions. While pretend play is often held up as an exemplar of children’s wild imaginations, and their abilities to take ordinary and everyday experiences and turn them into fantastical events, evidence suggests an alternative view: that young children’s pretend play is primarily grounded in reality, and serves the purpose of helping children anticipate unknown future real events (Fein, 1981; Harris, 2021). From drawing development to metaphorical reasoning, younger children (aged 3–5 years) are more likely than older children (8 years and up) to be grounded in reality and unable or unwilling to create fantastic alternatives to real worlds they know about. Young children (aged 2 and 3 years) often go so far as to reject non-realistic pretend play (Vondervoort et al., 2017), and children up to age 6 will often prefer realistic endings to stories compared to fantastical endings (Weisberg et al., 2013; Barnes et al., 2015). It is only later in development, with exposure to fantastical media and cultural stories involving elements of far fantasy (Goldstein and Alperson, 2020) that relatively older children begin contemplating the unreal.

Orientation to pretend play and fantasy is often conceptualized, operationalized, and measured as an individual difference in childhood (Bunce and Woolley, 2021) rather than being conceptualized and measured as a global, developmental trait. In preschool, a greater propensity toward play involving non-realistic elements is associated with stronger emotional regulation skills, over and above age or language skills (Gilpin et al., 2015) and affective empathy over and above cognitive theory of mind (Brown et al., 2017). Children who engage in more fantasy play, via imaginary companions or non-realistic role play, show higher levels of theory of mind (Taylor and Carlson, 1997) and fantasy play interventions seem to increase executive functions over non-imaginative play and other control conditions, with children showing the highest levels of fantastical play also show the highest gains in executive function (Thibodeau et al., 2016). The data, however, are limited primarily to preschool aged children, and assumes that pretense, pretend play and fantasy orientation drops off between the ages of 6 or 8 years (Smith and Lillard, 2012); the empirical literature has not meaningfully considered how older children and adults engage with fantastical, embodied pretense and imaginative play (Meyer, 2016).

Early developmental theory (Vygotsky, 1967; Piaget, 2013) conceived of pretend play as not necessarily serving a direct function itself, but rather as indirectly facilitating practice among children for important socio-cognitive functions in the real world. That is, play in fictive context *x* benefits action in real context *y*. At the individual level, these functions include physical and emotional control (Lillard, 2017; White et al., 2018), counterfactual thinking (Buchsbaum et al., 2012; Gopnik and Walker, 2013), creativity (Hoffmann and Russ, 2012), and factual and conceptual learning (Weisberg, 2015; Zosh et al., 2018). Pretend play also allows children to rehearse engagement with cultural institutions such as behavioral and object-directed norms (Nielsen, 2012), and formal institutions, including religion (Renfrew et al., 2017), which may be built upon evolutionarily derived selection pressures for survival (Steen and Owens, 2001), successful acquisition of artifact affordances (Nielsen et al., 2012), all via forms of natural pedagogy (Csibra and Gergely, 2011). Consider the perennial favorite game of pretense among Western children: *cops and robbers*. Here, children pretend to engage in behaviors that are consistent/inconsistent with cultural norms, to express physical and emotional aspects consistent with particular social schemas, engage in chase play, and to pretensively operate artifacts common to culture but beyond their immediate experience (e.g., such as weapons). While we are not claiming that pretend play *creates* those occupying undesirable social roles, such a familiar example neatly exemplifies that ways in which pretend play in fictive context *x* may inform real-world context *y*.

## Pretensive Shared Reality as an Extension of Childhood Pretend Play

So why might adult pretense exist in an ultimate sense? We suggest that adult pretense, specifically in the form of pretensive shared reality, is a spandrel of childhood pretend play. The evolutionary and biological definition of a spandrel is a feature or trait that did not arise for adaptive reasons, but which exists as a consequence of, or as a byproduct of, another feature of trait that *is* adaptive (Gould, 1997; Buss et al., 1998). Childhood play likely serves important and adaptive functions in childhood (Hirsh-Pasek and Golinkoff, 2006; Lillard, 2017), but is adult imaginative play adaptive? The strong response is “*no, it is not adaptive at all*,” while the weak response is “*no, it is not adaptive in the same way as it is in childhood*.” If the latter is true, then adult imaginative play has been *exapted* from childhood pretend play. While it is beyond the scope of the present article to determine if adult imaginative play is *adaptive* (Gould, 1997), we are comfortable in asserting – in response to the question *why does adult pretense exist at all* – that childhood pretend play arose before adult imaginative play (Nielsen, 2012; Morley, 2017), that adult imaginative play exists as a consequence of this adaptive developmental trait, incurs a cost (which is minimally a time-cost), and serves a different role in adult lives than in childhood. That said, while we softly reject the idea that adult imaginative play is fitness enhancing in adulthood as it is in childhood, we do accept that the many socio-cognitive functions adult imaginative play serves are similar to those of childhood.

## Interim Summary

Thus far we have attempted to establish the following premises:

1. *Adults do, in fact, engage in imaginative play. However this is not apparent if we judge what adults do by the standards of children.* Much as it is with children, adult imaginative play exists on a spectrum of being highly embellished with much pageantry, to being relatively spartan and minimalist. Adults engage in imaginative play during table-top role-playing games, live-action role-playing games, and some board games; when adults assume fictional identities during festivals such as halloween, cosplaying well-known figures in popular media at conventions, or engaging in some forms of kink-play; it occurs during “Model UN” events, Moot-court events, and historical re-enactments; imaginative play also includes “murder mystery” parties, “escape rooms,” and fantasy sports leagues, and may even include idle conversation about what one would do in a zombie apocalypse.
2. *Pretensive shared reality co-opts many of the socio-cognitive mechanisms of childhood pretend play, and serves similar qualitative functions as pretend play.*
3. *The key difference between pretend play and pretensive shared reality, however, is the greater reliance on shared intentionality and agentic expression which yields personal and social benefit under the umbrella term of* “*social utility*.”
4. *During pretensive shared reality, the pretend world box is cooperatively co-constructed, and player-character behavior therein* – *while governed by a set of rules about what is legal* – *is exceptionally rich in opportunities for embellishment and elaboration, as well as consistent and appropriate behavior*.
5. *During pretensive shared reality, each player cognitively quarantines belief-like states between themselves and their player-character, and while a certain degree of cognitive porousness exists between player and player-character, players operate with an understanding that game events are not* “*real*,” *but that game events can generate authentic cognitive, emotional and agentic experiences.*
6. The important feature of pretensive shared reality is certain permeability between the player and play, which is responsible for affective dimensions of the experience.

If we accept these premises then several implications follow. First, if pretensive shared reality is the qualitative extension of pretend play in childhood – even if it does have more implicit, explicit, and normative rules – then we can consider pretend play as serving a much larger role in life-history, and possibly even human evolution. Particularly with regard to the role pretend play serves in childhood in terms of supporting and creating various kinds of institutions (including religion) – a point developed by others (Nielsen, 2012; Renfrew et al., 2017).

A second, related, point is that it allows us to consider that, once children utilize shared intentionality within pretend play (Thompson and Goldstein, 2019), that the socio-cognitive functions of pretend play do not end when the child developmentally transitions to “rule based games.” Rather, that “social utility” of pretend play increases in maturity and sophistication from childhood into adolescence and beyond, to match the social and cognitive demands of more mature life-stages. Through this lens, research on the development of childhood pretend play is not simply an aggregation of cognitive milestones toward adulthood, but as one end of a spectrum along which various socio-cultural aesthetic experiences exist. Childhood is a period of delayed growth in favor of the acquisition of important social information (Nowell, 2016), but it is not the case that learning how to navigate physical and social environments ends with puberty – the demands simply expand to include many other, new, concerns, such as identity formation, higher-order representation of cultural and institutional obligations, and the maintenance of different forms of social relationships. Such a framework better incorporates the finding that children tend to prefer reality-based pretense, while adults tend to prefer the fantastic.

And third, if it is the case that pretensive shared reality is an extension of childhood pretend play, and serves many socio-cognitive functions, then it follows that pretensive shared reality is a central conceptual point along several axes of human culture, cognition, and behavior. As we will elaborate, the theoretical commonalities between exercises in shared reality may be represented as two-dimensional space in which two continuous features exist: physical embodiment and cognitive engagement. For example, watching the latest Marvel Universe blockbuster requires low physical embodiment (sitting in a theater), and requires only modest cognitive engagement^9^. Playing in an “Escape Room” generally requires more embodiment (physical manipulation of objects), and often quite substantial cognitive engagement (coordinated problem solving). Participating in improvisational or scripted theater requires considerable embodiment (styles of dress and appropriate movement) and cognitive engagement (the invention or recall of lines, an understanding of motives not belonging to the self which are to be communicated in performative ways). While participation in religious rituals may require various degrees of embodiment and cognitive engagement (Whitehouse, 2004). These dimensions and their implications will be elaborated hereafter.

Accepting the above, we also propose that pretensive shared reality, specifically exemplified by table-top role-playing games affords two empirical and research opportunities that are not presently addressed in the literature. First, examining table-top role-playing games as an organic phenomenon which can yield insights into various areas of psychological enquiry, and second, as a promising experimental paradigm with affordances beyond many existing paradigms.

## The Axes of Pretensive Shared Reality: Embodiment and Cognition

We propose that most kinds of imaginative cultures exist in a nebulous space that can be characterized by two axes: *embodiment* (externally, behaviorally relevant and observable behaviors) and *cognition* (complexity of the mental tasks). While we freely concede that this is a simplistic reduction, it is the case that such models can be useful (even if they may lack nuance, then again, “*fuck nuance*;” Healy, 2017). We accept that multiple other dimensions exist, though we anticipate that if one were to conduct a kind of factor analysis on the features of imaginative cultures, these two factors would account for the majority of variance, and themselves be relatively orthogonal.

Hereafter we will define our terms, but first let us revisit the work of Nichols and Stich (2000). Nichols and Stich (2000) argue that pretend play (in adults) requires the following four elements from the participants: establishing a premise, inferential elaboration, non-inferential elaboration (embellishment), and production of appropriate pretend behavior. It is clear that the first three points are cognitive, while the final point is behavioral. As is consistent with the developmental literature, pretend play in children is often difficult to define precisely, but is primarily determined through the *actions* of the child, in relationship to something *imagined* (e.g., hiding under the covers of the bed, because a boogeyman is in the wardrobe). This conception has precedent; our contribution is simply extending imaginative play to various other forms of imaginative culture, and in particular, pretensive shared reality. While our two axes conception is simplistic, we believe it is useful (as will be elaborated upon).

We define “embodiment,” within the context of pretensive shared reality (and imaginative cultures generally) as the degree to which the pretensive shared reality (or imaginative culture) *requires* the participant *to perform actions which are externally visible and behaviorally representative of, and consistent with, the pretensive shared reality with their physical body*. Table-Top Role Playing *requires* participants not simply to make agentic decisions, but to engage with those decisions via physical actions. Table-top role-playing games generally require fairly minimal embodiment, limited to speech acts and rolling dice. However, it is also quite common for players – when operating their player-character – to produce vocal affectations or accents, and even behavioral mannerisms or styles of dress that are representative of, and consistent with, their pretensive shared reality. Such actions are not required, but are usually encouraged, and are normatively permitted. This is in contrast to forms of imaginative culture which operate at either extreme of the *embodied* spectrum. At the low end there are imaginative cultures which *do not* require such behaviors: such as reading fiction, watching a movie, or listening to music. While at the high end there is religion and ritual practices, where behaviors are not simply required, but are prescriptive, defined in doctrine, and to which there is symbolic meaning associated with the actions (and often supernatural or moral considerations) (Whitehouse, 2004; Kapitány et al., 2020a; Nielsen et al., 2020).

We define “cognition,” in the same context, as the degree to which the experience *requires* cognitive engagement that is consistent with the object of shared intentionality (i.e., the pretensive reality of the group). Of course, no waking human activity is without active cognition, but some activities require “more” than others (while some may *arouse* cognitive engagement, they do not require active engagement). Again, table-top role-playing *requires active* engagement (as opposed to passive engagement). While it is possible that such engagement is minimal (e.g., to merely indicate that one’s player-character is going to swing a sword or shoot an arrow), it is frequently more active, it is still agentic. Players must create back stories and life histories for their player-characters, to hold motives which must be acted upon consistently in the world, and – most importantly – to engage in acts of agency consistent with the world (Nguyen and Thi Nguyen, 2020). Again, this is in contrast to forms of imaginative culture which operate at either extreme of the *cognitive* spectrum. At the low/passive end there are imaginative cultures which *do not* require [as much] active^10^ agentic cognition: reading fiction, watching a movie, or listening to music only require attention, but does not require cognitions associated with agency, problem solving, theory of mind, emotion regulation, or others; though it is important to note that such experiences may frequently arouse active cognitive processes, and doing so may be personally valuable or pleasurable, it is not obligate. While at the high/active end there are certain religious forms and ritual practices, where cognitions are not simply required, but are prescriptive, defined in doctrine, and to which there is symbolic meaning associated with the actions (and often supernatural or moral considerations) (Whitehouse, 2004; Kapitány et al., 2020a; Nielsen et al., 2020).

## What Questions Can Studying Table-Top Role-Playing Games Answer Directly?

Tabletop role-playing games are a specific and common example of imaginative play by adults. This topic is under examined by the psychological disciplines, and yet we believe that examining it directly can provide opportunities to answer questions surrounding cognitive engagement, the role of embodied- and social- cognitive skills, and understanding particular qualities of mind. Here, we suggest several low-hanging research topics related to pretensive shared realities like those apparent in TTRPG, such as D&D. We propose the following five research questions.

First, by examining how individuals and groups engage with pretensive shared realities in the context of table-top role-playing games we can ask questions regarding theory of mind and behavioral motivation. Shared pretensive realities require the explicit discussion of players’ reasoning, internal states, motivations, and desires. That is, the “belief box” associated with their player-character must be apparent to all players, and consistent with the pretensive shared reality, so that other players can accept and engage with it further. In real life individuals rarely explain their reasoning and motivation before they engage in behaviors. However, in table-top role-playing games, players must make the internal states of their player-character explicit to justify their [player-character’s] actions. In most cases, the action of the player-character need not necessarily benefit the group, but it must at least be accepted as consistent with the world. *Thus, the pretensive shared reality of table-top role-playing games can be used to observe, examine, and question topics associated with theory of mind, social justifications, shared intentionality and mutual understanding, by way of the often-necessary feature of making the implicit explicit*.

Second, the contents of shared pretensive reality can be used to illuminate and explore social problem solving. In such games, players must navigate themselves and their player-characters through various kinds of conflicts and problems that must be addressed in social ways. While social psychology attempts to examine social problem solving through a variety of tightly controlled and contrived scenarios, table-top role-playing games offer a more “naturalistic” opportunity to examine how groups of individuals negotiate outcomes within the confines of a variety of explicit assumptions, constraints, motivations, and contexts. Just like the real world, table-top role-playing games present players with challenges that [may] have zero-sum outcomes, which are morally or technically challenging, and which relate to the welfare of self and others. Contrary to the pursuit of an empirically “clean” and controlled laboratory environment, table-top role-playing games offer the opportunity to examine such questions in less messy contexts than the real world, but more elaborate and freeform environments than those of the lab. *Thus, table-top role-playing games can therefore be used to examine how groups collaborate around goals and form holistic solutions from disparate perspectives and abilities*.

Third, the collaborative requirements of pretensive shared realities can illuminate how shared realities are constructed, and to what degree certain counterfactuals and alternative agencies can be explored at the boundaries between *player* and *player-character.* Players may experiment with certain things that are safer, easier, and less consequential in a game-world than in the real-world. A simple example may be how a shy individual (player) may wish to exercise greater boldness or social competence, while a more multifaceted example would be for an individual to experiment with alternative gender or sexual identities. And while these may be trait-like qualities, certain alternative kinds of agency may also be explored: a law-abiding citizen may wish to exercise acts of violence, or a teetotaler may wish to play-act the conditions of addiction. *While many counterfactuals may only be trivially relevant to most individuals in general, the observation of table-top role-playing games in particular contexts can reveal how some individuals negotiate complex personal and social agencies*.

While our third example was primarily focused on how *individuals* explore various agencies in real time, our forth examples relate to how groups form, maintain, or change norms and institutions. Shared realities can be both fictional (much as they are in table-top role-playing games) or [normatively] real (like formal communities engaged in specific tasks). This includes examples as diverse as sports teams with the shared goal of scoring more points than another team, but may also include moral communities relating to social, political, or religious ideologies. In such cases, the expectations of one’s inner motivations must be reconciled and/or delineated from the “real” world, and must be agreed upon to an extent that each member of the group “buys in” to the set of terms regarding what is acceptable or forbidden. This may be trivial, such as what body part may/may not touch a ball, or profound, such as whether or not violence is permissible as a means to an end. These shared realities only work when all members of the group follow the internal rules and boundaries. Consequently, breaking these can ruin the coherency of the group (leading to a dissolution of social or personal identities, or schisms within larger groups). *Table-top role-playing games provide low stakes and delineated opportunities to examine and interrogate how individuals constitute groups in real time as a function of invented norms and institutions*.

Our fifth suggestion is that table-top role-playing games are opportunities to examine microcosms of meaning-making (by both individuals and groups). When individuals navigate a pretensive shared reality with a player-character that is distinct from their own identity, we can examine how they [the player] may understand their own behaviors and desires [in the real world] through the lens of their player-characters’ behavior and desires [in the game world]. An individual may never have considered, for example, whether or not they had a desire or capacity for self-sacrifice, but through the game world, they may be confronted with such a choice. *In table-top role-playing games and other realities, individuals can construct identities and narratives that go beyond their everyday lived experiences, thus allowing for examination of choices that may not be available in the real world, and a construction of an understanding of one’s own behavior that is not always apparent outside of a separated or quarantined “reality.”*

Our sixth and final suggestion utilizes Nguyen’s conceptualization of games as a medium of agency and portrays role-playing games as a possible laboratory for the study of agency itself, that is as a medium of participatory self-making. In the spirit of enactivistic models of cognition (De Jaegher and Di Paolo, 2007; Barandiaran et al., 2009; De Jaegher et al., 2010), *role-playing games seem to be fitting examples of information ecologies strongly dependent on iterative scaffolding of perception-action loops* – *the meaning-making is participatory and its parties are strongly co-constructed in the process. The characters come alive through play, they seem to obtain at least partial autonomy from the agency of the players and as such form interesting phenomena for the psychology of self.*

While this is far from a comprehensive list of research opportunities, we hope that greater scholarly attention will be given to pretensive shared realities, and specifically table-top role-playing games such as D&D. Table-top role-playing games are popular, relatively easy to learn, require as much (or as little) researcher-input as desired, occur naturalistically and organically, and capture a wide diversity of topics that interest behavioral scientists: all of which marks them out as an underutilized topic of inquiry. They also seem to be less costly than live-action role-playing games in terms of embodiment and commitment, D&D is very much comparable to board games, i.e., there is still quite visible distance between player and character which in LARPs diminishes when the table disappears to physical world and players need to fully embody their actions.

## Table-Top Role-Playing Games and Pretensive Shared Reality as a Research Paradigm

While the discipline of “psychology” is diverse, we believe that various sub-disciplines involving basic research on social and social-cognitive topics, may benefit from engaging with TTRPG-like methodologies that rely on pretensive shared reality. We must be clear, however, that we are not suggesting this is some kind of methodological magic bullet – far from it – but simply that there are some instances in which embracing the assumptions we’ve outlined *may* have greater claims to validity and generalizability. We have identified three broad conditions where methodological innovation may be possible if researchers utilize insights derived from our outline of pretensive shared reality. We will provide one example from the field of Moral Psychology which we think encapsulates our argument.

Our first condition is pragmatic. There are many domains of study – particularly experimental study – where researchers cannot ethically or practically administer a manipulation. We suggest, in principle, that most studies that rely on vignettes can be improved by embracing greater complexity via pretensive shared reality.

Our second condition relates to researchers acknowledging their own assumptions. Again, researchers that use vignettes or priming paragraphs are asking participants to semantically or affectively embrace a set of premises, then act in some responsive way. Creating more complex and immersive scenarios may produce more valuable focal outcomes.

Our third condition relates to experimental demands. In many kinds of research participants may respond in socially desirable ways. By creating a clearer delineation between the participant and the scenario of interest, participants may be less likely to view the behavior as diagnostic of their own inner-states.

Hereafter, we must offer some caveats: first, while we believe we have identified (as many others have before us) that there are various practical and pragmatic short-comings associated with the methods of experimental psychology, our suggested responses are speculative. Too little research has examined this domain for us to consider these as more than suggestions. And second, we must re-iterate that pretensive shared reality is not the same as unconstrained childhood pretend play. Our conception of pretensive shared reality as the co-operative co-construction of premises and conclusions performed by adults in imaginative and relatively unconstrained ways. As with most psychological constructs, it is a spectrum. There may be more or less co-construction, greater or fewer constraints, and richer or more impoverished imaginings involved. Again, this is not a methodological magic bullet, but a possible avenue of innovation that *may* yield results that are more valid or generalizable than existing methodologies. We foresee two general caveats of using role-playing games as projective research methodology: (1) the richer ecological validity inevitably leads to lesser control and (2) the social role-playing games are to some extent dependent on several social skills, which introduces its own biases.

Moreover as role-playing is in some manner unconstrained complex creative activity, the participants could and would follow orthogonal individual heuristics for making sense of the situation and the role of their own agency within the play if they are not clearly instructed/conditioned by the experimental design. For example players could implicitly understand their playing agencies along different aspects of the collective action (cf. Edwards, 2001; Torner, 2018), e.g., as gaming (“we are supposed to compete”), or storytelling (“we are supposed to co-create and enact a story with a theme”) or simulation (“we should try to realistically portray the situation”). These heuristics bring their own possibly skewing influences on the individual and collective level for which the research design needs to counterbalance or use them for the purpose of the research. As our primary example let us consider an area that touches on multiple research interests of behavioral scientists, and which is widely known. Let us consider how experimental psychologists and philosophers examine moral decision making: an area that has multiple pragmatic and ethical concerns, where experimental demands are extensive, and where acknowledging researcher- and participant-centric assumptions are core.

The classic trolley problem (Foot, 1967) is typically presented to participants as a vignette, because asking undergrads to literally kill people is neither ethical nor practical (condition 1). According to a relatively standardized protocol (Greene et al., 2008), participants are briefed about what to expect, which also includes disclaimers that aim to alleviate socially desirable responding (condition 3): “*Moral judgments can be difficult to make, and we understand that people sometimes change their minds about moral questions or feel conflicted about the answers they’re given. Don’t think of your answers as “written in stone.” All we want from you is a thoughtful first response”* (Greene et al., 2008). Participants are then presented with a options they must report as acceptable or not^11^. Arguably, the most famous example of a moral dilemma is a variation on the “trolley (switch)” problem, known as the “Footbridge” problem (with or without reference to a “fat man”). Thomson (1985) originally described the problem in philosophical terms (rather than experimental):

*Consider a case*… *in which you are standing on a footbridge over the trolley track. You can see a trolley hurtling down the track, out of control. You turn around to see where the trolley is headed, and there are five workmen on the track where it exits from under the footbridge. [.] It just so happens that standing next to you on the footbridge is a fat man*…. *He is leaning over the railing, watching the trolley; all you have to do is to give him a little shove, and over the railing he will go, onto the track in the path of the trolley* [and prevent the deaths of the five workers]. *Would it be permissible for you to do this?*

Before we consider whether there are assumptions here with regard to pretensive shared reality, let us first consider the paradigm in terms of Nichols and Stich. Is there a pretend world box (or, a set of premises)? *Yes*, in the first instance a “workspace” is proposed by the researcher: it involves a footbridge, a large man, a trolley/train, and five workers. Does the participant have a meaningful belief box (or, a willingness to make inferences about the world). *Yes*, they simply must assume that the lives of these entirely fictional, nameless, identity-less individuals matter, that their deaths in some sense represent death as it is in the real world (Majdandžić et al., 2012) (participants are also told explicitly a rather unlikely additional premise exists: that one overweight man is sufficient to stop a rolling train, while a “little shove” is enough to put him over the railing). Is there non-inferential elaboration – or embellishment – in the scenario? *No*, this is typically forbidden by the protocol. Participants cannot, for example, ask whether the large man has a family, whether the workers will notice the train at the last moment and save themselves, whether intercessory prayer is an option, or if this is a world in which it is customary to execute workmen by locomotive. That said, participants *want* to ask these questions, and there is emerging evidence that participants are embellishing the situation in imaginative ways, whether the researcher wants them to or not (Hauser et al., 2007). And so while there is no formal embellishment, many participants engage in these practices privately. Meanwhile, within the study of moral decision making, the debate rages as to whether such minimalist designs have external validity (Bauman et al., 2014). Finally, is there production of appropriate pretend behavior? *Yes*, in the sense the participant makes evaluation as a speech act or a mark on a page consistent with all that has preceded it (rather than, say, disengaging with the question and simply leaving the room). And so we see that the minimal conditions are, to greater or lesser degree, apparent to meet the definition of pretense advanced by Nichols and Stich (2000).

Perhaps more important in this discussion is the concept of cognitive quarantine. In the above example, a participant operates some kind of player-character of themselves. They must operate the player-character consistent with the assumptions of the pretend world (i.e., that the body of a large man may stop a train, and that the workers’ lives matter). The cognitive quarantine under such conditions is remarkably porous: not only do participants self-report an emotional experience, it is, expectedly, observable in parts of the brain associated with emotional engagement and processing (Greene et al., 2001). This, alone, is not remarkable. What is striking is that in instances where the situation is presented in virtual reality (VR), where there is some sort of merging between the participant and the player-character (Francis et al., 2016, 2017)^12^, there is greater porousness (with regard to reported emotions and physiological arousal; Francis et al., 2016; McDonald et al., 2017) *and* that, on average, more participants perform the act of sacrificing the large man (Francis et al., 2016, 2017). Questions of external validity relative to vignette studies have long been raised (Bauman et al., 2014; Bostyn et al., 2018), however, we maintain that an awareness of the porousness of the cognitive quarantine can be exploited in order to generate new knowledge. And as such, this topic meets our second condition, that there may be unacknowledged assumptions inherent to the work.

The domain of moral decision making generally meets the conditions in which table-top role-playing game-like methodologies may benefit empirical research. First, there are pragmatic and ethical and practical concerns. One cannot literally give a participant the power of life-and-death. Second, there are researcher-assumptions: What is the moral worth of a “fat man” on a bridge, or of “five workers”? (This has long been known to researchers in this area). Third, there are experimental demands that influence focal outcomes. Again, this has long been known, but as evinced through VR methodologies, it is clear that creating richer experimental environments is valuable, and to the extent that VR offers insight, we argue that so too can TTRPG-like methodologies. Finally, it is clear that this area meets many of the classic conditions for pretense as described by Nichols and Stich: (a) shared sets of premises, (b) inference, (c) elaboration, and (d) pretense-consistent action (whether they are explicitly acknowledged or not). Thus, embracing the assumptions outlined here in terms of pretensive shared reality, and in particular cognitive quarantine, may make room for methodological innovation and advancement.

## Examining Imaginative Cultures Through the Lens of Pretensive Shared Reality

We now arrive at the most contentious aspect of our argument: that our understanding of pretensive shared realities not only illuminates play, games, and the obviously fantastical, but can also illuminate our understanding of social institutions with imaginative culture components, like religion. We need to be clear: we are not making the claim that religions are simply “made up,” “fantastical,” or the product of “pretense.” Our term “pretensive shared reality” was defined in Section “Imaginative Play as “Pretensive Shared Reality,” but can be summarized in the following way: *Pretensive shared reality describes a space of mental objects of shared intentionality, that is durable, and which embodies a set of (implicit or explicit) premises, rules, or norms that knowingly differ from the “real world” by some degree; pretensive shared reality permits and constrains the behavior of both players and their player-characters (consistent with the premises and/or norms of the imagined world)*. Meanwhile, we accept the definition of imaginative culture as *shared and transmissible mental experiences that are aesthetically and emotionally modulated*. Thus, pretensive shared reality is neither superordinate nor subordinate to this concept, but something that explains a subset of imaginative cultures.

We argue that this definition makes room for religion, in the sense that the epistemic qualities of religious claims are shared, durable, and embody rules. We do not care to weigh in on whether religion describes reality, but only wish to demarcate the epistemic quality of the claim “God is real” as different from the claim that “this table is real” (inasmuch as the way I can directly interact with a table is different from how I may interact with God). Moreover, we do not wish to equate the religious entity *God* with a table, or any hypothetical fictive entity. We only need to maintain that such targets are shared by a community, are associated with various kinds of norms, require certain kinds of behaviors, and have an enduring quality. The final point is most important; any form of imaginative culture, from games to religion, requires a degree of sharedness; any individual human can engage with their own imagination and its products, though we would hardly talk about culture if it was without a dimension of sharedness and engagement. Further, we argue pretensive shared reality relies upon two axes: embodiment and cognition. Religion can be characterized along such dimensions (Whitehouse, 2004; Kapitány et al., 2020a; Nielsen et al., 2020). Meanwhile, many researchers preceding us have argued that childhood pretend play is evolutionarily related to religion (Nielsen, 2012; Morley, 2017), and here, we extend their arguments further than previously committed to text, by examining what – exactly – are the constitutive parts of pretensive shared reality.

What is important in our claim is not whether religions are pretensive, but rather, whether our conception of pretensive shared reality can further illuminate the pursuits of the cognitive sciences of religion (CSR; Xygalatas, 2014), and whether examining religion can shed light on the study of the imaginative capacities of adults. We assert that there is a phenomenological “kinship” between pretensive shared reality and religion, and believe that insights from one can inform the other. Further, we suggest that experimental investigation into topics associated with religion (such as how beliefs are formed and maintained, particularly in unseen non-physical entities) can benefit from incorporating TTRP-like experiences.

Before we defend these points, let us consider religion in the terms of psychology and the cognitive science of religion. Humans seem to be evolutionarily hardwired for social behavior and have a strong capacity for “shared reality” (Echterhoff et al., 2009). Yet it cannot simply be assumed that any random collection of humans will generate a shared reality. Such a thing would need to be established through communicative interaction and would be dependent on multiple factors (some of which we have discussed, some we have not). A shared reality must necessarily be largely based on the ability of individuals to exchange information, the acknowledgment of individual goals and values, and require both a capacity for individuals to form group goals, and a catalytic incentive or pressure for the interaction. The most relevant theoretical consideration between pretensive shared reality is the ways in which the sharedness is created, and the ways in which social identities are constructed. The definition of a ritual, according to Hobson et al. (2018) is that rituals are *(a) predefined sequences characterized by rigidity, formality, and repetition that are (b) embedded in a larger system of symbolism and meaning, but (c) contain elements that lack direct instrumental purpos*e (p. 261). Pretensive shared reality overlaps considerably: within a pretensive shared reality (a) norms define what can/cannot be done, (b) claims and behaviors are embedded within a larger system (i.e., the pretend world box), and (c) behaviors are not instrumental [to the real world]. While we are careful not to equate “rituals” with “religion,” many scholars within the cognitive science of religion argue that rituals are what unites religious communities in a practical sense (Whitehouse, 2004; Atran and Henrich, 2010; Rossano, 2012; Norenzayan et al., 2016; Renfrew et al., 2017; Hobson et al., 2018; Kapitány et al., 2020a; Nielsen et al., 2020). Shared pretensive reality – in the form of table-top role-playing games – and ritual/religious behavior both require a strong institutional frame (that is, a set of agreed upon premises), which establishes a basis for shared intentionality and individual engagement with representational content therein, and both require behavior consistent with the stated premises. While this content (in both table-top role-playing games and religion) is usually counter to physical “reality” by various degrees, it becomes a pretensive reality when it is shared, and elicits agentic action.

However, it is important that we examine in what ways “reality” is conceived of as an object of shared intentionality for various groups. Luhrmann (2020) argues “*that god or spirit— the invisible other— must be made real for people*.” If we accept that “the invisible other” (whether God, a deity, or some non-agentic force) can be equated to a player-character (or any other enduring aspects of a pretensive shared reality) we can use Luhrmann’s framework to further our argument. God(s) are not empirically or directly observable, nor are game worlds. Such objects require us to accept certain premises, engage in inference and elaboration, and act with consistent behavior. In this sense, the unreal becomes real through cognitive and embodied engagement; invisible others become real through experiential mechanisms (whether rituals, or imaginative, inferential and elaborative game play) and are made experientially real through the expression of agency in counter-factual realities, whether invented by our GM, or described by a religious authority.

But a serious objection arises: It seems the case that the reality of the spiritual is differently real than the reality of everyday observable objects, like tables, and different from fictional entities, like Bishbosh the Goblin Shaman. Yet the core distinction between spiritual (and fictional) objects and profane objects is that the latter do not require “*any effort to experience them as rea*l” (p. 5). Unlike tables, which we simply accept as real, the contents of a pretensive shared reality must be established, inferred, elaborated, and require action. Religions are not obviously real. While the debate continues as to whether children may be natural theists (Kelemen, 2004) or not (Banerjee and Bloom, 2013), what is obviously true is that even if naive individuals have theistic tendencies, it’s less than likely that such tendencies will produce a consensus of theistic doctrine, or consistency in ritual action (Kapitány et al., 2020a). As such, the contents of religion must be established, inferred, and elaborated upon, much as the contents of a pretensive shared reality must also be. In each case we must construct legitimate beliefs (or belief-like states) for non-observable claims. As a result there is a similarity between the processes that establish and maintain [belief-like states for] Bishbosh the Goblin Shaman and spiritual figures, which – importantly – are different from how we individually establish a belief about a table. These are effortful experiences, and through expression of agency and cognitive effort, as well as embodied (and often ritualistic actions) belief-like states and shared intentionality and created and maintained (Kapitány et al., 2020b; Luhrmann, 2020). And due to the complexity of evaluating reality-status claims, adults tend to assign epistemic tags to their knowledge (Harris et al., 2006) which are categorized within various domains (Harris and Corriveau, 2021).

This reference to the general human capacity of operating on different ontological levels and its intuitive connection with action brings us back to the concepts of mechanisms like cognitive quarantine. The status of non-obvious things needs to be socially constructed, recognized, and attributed to its proper epistemic level and domain. Thus, we arrive at a response to the concern that the motives for imaginative play and imaginative cultures are distinct from religious motivations: Pretensive shared realities generate social utility, a process that may include generating personal and group-level enjoyment or mirth, the creation or maintenance of social groups, or the safe exploration of individual self-concepts. Social Utility, under the umbrella of pretensive shared reality and table-top role-playing is normatively co-operative and not interpersonally competitive. Pretensive shared realities are cognitively quarantined. In religious practice, people usually strive to enmesh their religious practice to their daily life, or for the sake of the daily life, because it feels rewarding, creates and maintains social groups, and allows for the development of both social and self-identities. Religious practice, like pretensive shared realities, are constructed in a special domain with special behavior. The primary distinction in this active meaning-making is the degree to which the participants seek durability in the belief-like states, and the degree to which the belief boxes are cognitively quarantined (games are highly quarantined – Stenros, 2014 – while religion is not).

If there is a significant overlap of social and cognitive mechanisms for shared reality between religious ritual and ritual as some authors propose (Whitehouse, 2004; Atran and Henrich, 2010; Rossano, 2012; Norenzayan et al., 2016; Renfrew et al., 2017; Hobson et al., 2018; Kapitány et al., 2020a; Nielsen et al., 2020), it seems apparent that religion with its more-serious motivation is simply (and desirably) more porous. In both pretensive shared reality and religion, spaces for specific behavior exist, and a boundary for cognitive, affective, and agentic experience exist, and which broadly serve the same goal of social facilitation. And as established in the previous section, some domains of research may generate greater insights by experimentally applying TTRPG-like methodologies. Here, we suggest that conceptualizing religion as a non-special case of a shared reality (pretensive or otherwise) allows for important insights, methodological, historical, and psychological. And while many scholars within the field of cognitive science of religion are likely to accept these claims, drawing a direct line between childhood pretense, adult pretensive shared reality, and religion, may still be fruitful.

## Limitations, Unexplored Opportunities, and Future Directions

We have attempted to outline a relatively simple thesis. Contrary to the common wisdom, adults do engage in imaginative play (much like children) and it is remarkably common. This play can be encapsulated within our proposed definition of “pretensive shared reality.” A key example of pretensive shared reality is table-top role-playing games, like D&D. These are theoretically, practically, and epistemically interesting because they are popular, require very little training (for participants/players), have no functionally upper-limit on descriptive richness or opportunities for embellishment, and are exceptionally flexible. We have contextualized our arguments in relation to the seminal work of Nichols and Stich (2000), Piaget (2013), who have (among others) shaped our understanding of these topics. We have (humbly) extended their work by elevating the importance of the social sharedness of this phenomenon, and described both the individual and evolutionary reasons why adult imaginative play is common, important, and interesting. We outline why the study of pretensive shared reality, specifically in the case of table-top role-playing games, is interesting in-and-of itself, how it may be used to generate further methodological insights, and how this framework (conceptualized along the axes of embodiment and cognition) can allow for a reconceptualization of existing work. However, we acknowledge many limitations.

First, this manuscript is but a foundation for further work. It is neither complete, nor comprehensive, though we hope it is sufficiently broad and clear so as to direct further thought. We have focused on the relatively “dry” topics of embodiment and cognition, while deliberately avoiding discussions associated with topics such as emotion expression and intensity. For example, how does cognitive quarantine and the porousness of belief create and/or interact with the generation and expression of emotion? Can TTRPG-like methodologies be used to study affect? In what way can affect be manipulated in order to influence decision making under conditions of pretensive shared reality? We have only briefly discussed questions associated with participant agency. To what extent does agency in a pretensive shared reality meaningfully correspond with a sense of agency in the real world? Emerging research suggests that game-like methodologies produce valid measures of personality, and overcome various issues associated with participant motivation and demand-responses (McCord et al., 2019), and we anticipate this to be true in other domains (though much validating work is required). We have not discussed in any depth important discussions in the psychological study of acting and aesthetics, particularly with regard to whether participants/players inhabit their player-characters authentically, or merely adopt the “mask” of their player-character (Appel, 1982). We have given no time to the individual differences associated with role-playing or transportation. Fantasy Orientation (in childhood) is widely recognized to be an individual difference (Bunce and Woolley, 2021), and the same is true in analogous constructs in adulthood. How, then, are we to integrate such work into pretensive shared reality? Nor have we empirically dissected “sharedness,” and the degree to which individuals lead (or conform to) decision making in group contexts, or the degree to which a social identity may be constructed within a pretensive shared reality. And finally, we have not discussed “reality,” and the degree to which pretensive shared realities meaningfully correspond with (or deviate from) the real world. Nor have we attempted to quantify degrees of pretense: are some kinds of deviation more or less accessible to participants, do different degrees of deviation impose greater- or lesser- cognitive load (and consequently influence the degree to which the pretensive reality is shared or the degree to which participants commit to it). We lament our inability to pursue these lines of enquiry and discussion, due both to time and word constraints, as well as the limits of our own expertise. However, we hope to have allowed sufficient room for future discussion. We invite contact and collaboration.

We have, however, outlined some key ways we believe adoption of this framework can benefit psychological enquiry generally. First, pretensive shared reality as expressed in the form of table-top role-playing is an understudied and interesting topic in its own right. Second, applying TTRPG-like methodologies may yield additional insight. Third, although such methodology could be fully independent from its original sources (like D&D), it cannot be overlooked that still the strongest knowledge about the role-playing games’ causes and effects lies in the ethnographic evidence and theorizing of role-playing studies, i.e., any crafting of such methodology would greatly benefit from a collaboration with them. And fourth, taking the concepts herein, we may recast our understanding of various developmental and evolutionary topics: in particular childhood pretense. We hope our work has been sufficiently self-apparent and careful in defining terms, limiting the boundaries of what we can (and cannot) claim, and suitably (but not exceptionally) provocative. Though it is a trope to call for “further research,” we stand firm that this manuscript is the foundation for our own further research, and we hope others are persuaded by our argument.

## Conclusion

Adult imaginative play is common, sophisticated, and an interesting model for understanding a wide variety of topics of interest to behavioral scientists, including social topics (such as social identities, norm formation, and group problem solving), cognitive topics (such as theory of mind, meta representations, and pretense) and developmental topics (specifically the longitudinal and ontological development of pretense throughout the lifespan). Though long standing definitions of childhood pretend play have largely rendered adult imaginative play empirically invisible, it is a topic worthy of considerable investigation. We have examined table-top role-playing games (and specifically D&D) as we believe they stand as a simple exemplar of many of the qualities most interesting within the domain of adult imaginative play, though we explicitly argue that they are but one example among many. We have not only outlined why this is a topic worthy of study, but how application of TTRPG-like methodologies may benefit behavioral scientists, when their work is pragmatically or ethically challenging, when participant demands are high, or when there may be hidden assumptions buried within standard methodologies. We have also (somewhat contentious) argued that further study on pretensive shared realities can shed light on cross-culturally interesting topics such as morality and religion. We make no claim that what is in this manuscript is entirely comprehensive and exhaustive, but we do hope it may serve as a foundation for future research, and we welcome critique or opportunities for collaboration.

## Author Contributions

All authors listed have made a substantial, direct, and intellectual contribution to the work, and approved it for publication.

## Conflict of Interest

The authors declare that the research was conducted in the absence of any commercial or financial relationships that could be construed as a potential conflict of interest.

## Publisher’s Note

All claims expressed in this article are solely those of the authors and do not necessarily represent those of their affiliated organizations, or those of the publisher, the editors and the reviewers. Any product that may be evaluated in this article, or claim that may be made by its manufacturer, is not guaranteed or endorsed by the publisher.

## Footnotes

1 For the sake of clarity when discussing children we will use the term “pretend play” (consistent with the literature), and when discussing adults we will use the term “imaginative play.”

2 In this paper we largely omit kindred forms of adult role-play, especially that of LARPs (live-action role-playing games) which almost deserve a stand-alone argument, as they include even stronger participatory embodiment than tabletop games. See Harviainen et al. (2018).

3 Though we acknowledge that the study of performing arts and acting use the phrase “living truthfully under imaginary circumstances” (Meisner and Longwell, 1987).

4 The notion of aesthetic motivation connects the experience of role-playing to the experience of art, but also is probably a theoretical step away from the clear conceptualization of the problem (what is the function of “aesthetic” in a psychological sense?). There is classical account of personal player motivation in RPG theory (Edwards, 2001; for broader discussion see Torner, 2018), which distinguishes three ideal types of players and playing styles: gamist (enjoys and focus on the “game,” stresses competition), narrativist (enjoys focus on “story,” stresses creativity and storytelling within a theme, genre) and simulationist (enjoy experiential consistency of play; stresses enactment of play with internal logic and consistency), from which the latter two could be understand as aesthetic motivation.

5 We use the terms “adult imaginative play” and “shared pretensive reality” in a functionally interchangeable way. Shared Pretensive Reality, however, is but a subset of all possible forms of adult imaginative play. We generally prefer the latter term in order to make room for scholars who wish to discuss “adult imaginative play” in ways that differ from our own, but will sometimes user the broader term to refer to the broader category of action.

6 Not to be confused with “social utility functions” (commonly referred to simply as “social utility” by Loewenstein et al., 1989). Their definition of a *Social utility function* is that of the consequent level of satisfaction as a function of outcome to self and others in a social decision making context.

7 Though it is unclear what affordances exist when pretending to be a dead cat. We suppose one might engage in the process of dying, or twitch, or take on a static and disfigured pose… but these are beyond the specific affordances of the premise (as would pretending to be the ghost of a cat, or some kind of undead cat).

8 For a lengthier discussion about the explicit and implicit power structures within the context of D&D rules, Garcia (2017).

9 We are not attempting to belittle fandom of this genre/franchise. Certainly, fans may engage with some topics, themes, and the universe at large in a very elaborate and cognitively complex way. However, one is not *required* by the circumstances to do so. Also the social cognition needed to reconstruct even the main axes of social complexity of the fictive world is scaffolded by the narration. Moreover the social order is fixed, while in social games it is in the making.

10 Active denotates here a possibility or necessity to engage with a decisive change of or within the imaginative situation.

11 We note that there are multiple and varied ways to do this, and not all scholars follow the same protocol.

12 The exact nature of this is unclear and hotly debated, as is the distinction between “moral decision making,” “moral action,” or “imaginative salience.” The specifics are less important than the broader point, however, that some kind of self-player-character merging is clearly occurring.

## Appendix A

### Basic Outline of How Dungeons & Dragons (Fifth Edition) Is Played

The following is a description of how Dungeons & Dragons (D&D) is played. D&D has been chosen as the example as it is the most famous and most influential form of table-top role playing, even though many diverse examples exist (some of which bear little resemblance to D&D). While the following description is likely to seem superficial to anyone familiar with the rules, the specifics are not as important as the general, illustrative features.

A game usually consists of one to many sessions, which take several hours of real time. A typical setup is “campaign play,” which takes the players over a series of predesigned narrative and character developments. A D&D campaign usually begins with a Game Master (GM) organizing a group of players into a group, which involves collective decisions about the nature of the game and creating or purchasing (in book or pdf form) a game-world and an adventure therein. The adventure may have next-to-no narrative (*explore a dungeon and secure the treasure*), or have a rich narrative (employing many fully fleshed characters with independent motives, relations, and conflicts), or anything in between. What’s important is that the GM presents some goal for which the players can share intentionality. The GM role has multilayered playwriting and performative responsibility, on one side they must design/prepare/improvise the story-arc or plot (*This will be a story about group adventures rescuing a beautiful dragon from an evil princess*). The GM also sets the environment and time-frame, and manipulates the narrative space. For example, a GM may set the scene (*You find yourselves in a tavern, there is a musician on a small stage singing. Around you tables are full of others appearing more or less reputable than yourselves. A fire-place heats the low-ceilinged tavern, and the room smells faintly of old beer)* and then allows players to choose [any] actions; the GM asks players to roll dice to determine if those actions are successful.

Each player arrives at the game with a player-character. While each character may have more or less backstory (depending on the disposition of the player, or the expectations of the GM), each character *must* have a set of abilities – statistics for game mechanism predetermining the probabilities of outcomes for specific encounters. In D&D there are many abilities, but they are all governed by six basic “skills:” *Strength, Dexterity, Constitution, Wisdom, Intelligence, Charisma*. Such skills are represented by a number between one and twenty, corresponding to a positive ability to perform skills, or negative ability. If a player wishes to do something, it is governed by a skill/ability. If a player says, for example, that they wish to *stand on the stage and perform a song, in order to create a distraction for the thief character to steal money from the bar*, then the GM will requires a skill check (in this case the performing character is likely to perform a “performance” check which is governed by *charisma*, and the thief character is likely to perform a “sleight of hand” check which is governed by *dexterity*). A check involves the DM determining how difficult a task is, then asking the player to roll a 20-sided dice (and then to add their modifier). The “performance check” is usually a number from 1 and 20 (though higher numbers are possible). Higher numbers represent a more challenging task. The GM may decide entertaining a tavern full of people who are drinking is relatively easy (a challenge of 12) but stealing the money is difficult (a challenge of 18).

Perhaps the would-be performer has a negative modifier. They roll, say, a 15, and then add their modifier (a negative 2). They scored 13, which is higher than 12. *The room is enthralled* (At this point, the GM may ask the player to describe the scene, or the GM may describe it themselves). Then the thief character rolls their check. They rolled an 8, but since they are good at theft, their modifier is +6. Their total, 14, is lower than the challenge rating. The thief fails. The GM would usually describe the scene: *As the singer stands on stage singing a captivating song about a long past lover, the thief moves inconspicuously toward the bar. With a small act of misdirection, and confident in their ability to grab a handful of coins, the thief reaches into the till*…. And it is here the act fails. It is now up to the GM: does the bartender grab the thief’s hand and threaten to call the guards? Does the bartender notice the act, and let the thief keep the money, with the intention to blackmail the thief later? Does the bartender cast a spell and put the thief to sleep? The GM will usually make a decision that makes the world richer and adds value and motivation to whatever shared goal the group is pursuing. Importantly, whatever the GM chooses to do, is constrained by certain acts that are legal within rules of the game and consistent with the premises, but which have exceptional degrees of freedom. All three examples described above may be legal and consistent, but hundreds of alternatives exist.

Though this is a brief and impoverished description of how D&D is played, it can be put into any imaginable setting, and include a wide array interactive elements (many of which are described in rule books, where the rules are explicitly acknowledged as guidelines under the discretion of the GM).
